# Highlight: Cellular Environment and Protein Structure Help Organisms Cope with Temperature Stress

**DOI:** 10.1093/molbev/msaf155

**Published:** 2025-07-15

**Authors:** Pedro Andrade

**Keywords:** protein, thermal stability, yeast, evolution, temperature, adaptation

The molecular pathways that organisms use to adapt to their environment can be as varied as the environment itself. However, a few environmental factors pose consistent constraints on adaptation across domains of life. Temperature is one such ubiquitous factor. It affects organismal function across multiple scales, including cells and their molecular building blocks. Protein function and stability, for example, are heavily influenced by thermal conditions, in particular a protein's ability to adopt its native 3D (folded) configuration (Pucci and Rooman 2017). Since proteins are the cellular effectors of genomic information, studying how temperature affects protein stability holds great promise for dissecting the cellular and molecular mechanisms underlying adaptation to thermal stress. However, a protein's thermal stability can be influenced by its amino acid sequence itself, as well as the broader cellular environment; separating sequence-based and cellular-based effects on thermal stability has proven challenging.

Justin C. Fay, a professor at the University of Rochester in the United States, has been working on the evolution of thermotolerance in yeast (*Saccharomyces* spp.) for some years. Recently, an opportunity presented itself to dig deeper into this field: “Around 2017, when my lab moved to the University of Rochester, we were taking a genetic approach. Our colleague Sina Ghaemmaghami made the great suggestion of taking a biochemical approach using thermal proteomic profiling, with which people had shown rather dramatic differences in stability between mesophiles and thermophiles.” For Fay, this suggested that divergence in protein stability could also occur between closely related species, including the species of yeast, which vary considerably in their thermal optima (Fig. 1). Fay adds “We took Sina's suggestion and gained a lot of traction with his help on the proteomics and help with protein purification with my colleague Eric Phizicky in the Biochemistry Department.”

**Fig. 1.**
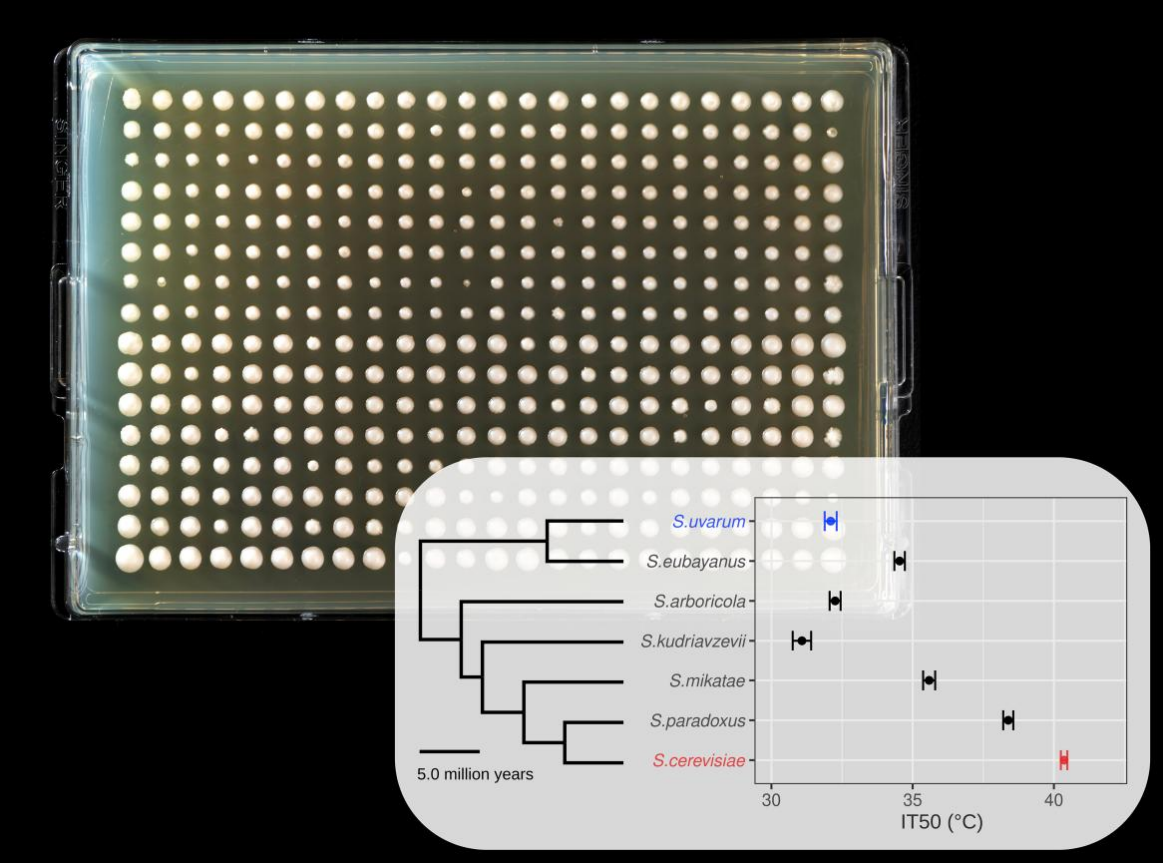
An example of the agar plates used by Walunjkar et al. (2025) to conduct yeast phenotyping assays at different sets of temperatures. Authors focused on the species *S. cerevisiae* (at the bottom of the plot) and *S. uvarum* (at the top of the plot), which possess a large difference in their optimal temperatures following 16 million years of evolutionary divergence; however, these species can still produce viable hybrids. Agar plate photo by J. Adam Fenster/University of Rochester; inset plot by Walunjkar et al. (2025).

A new article, now published in *Molecular Biology and Evolution* (Walunjkar et al. 2025), shows how the collaborating Fay–Ghaemmaghami–Phizicky labs devised an elegant solution to disentangle sequence and cellular effects on protein thermal stability. Walunjkar et al. worked with two yeast species, *Saccharomyces cerevisiae* and *Saccharomyces uvarum*. These have a 16-million-year history of evolutionary divergence, during which they have evolved starkly different thermal optima, with *S. cerevisiae* having an optimum temperature that is nearly 8 °C higher than *S. uvarum*. Crucially, these stark differences in phenotype do not prevent these species from producing viable hybrids, providing these scientists with a great opportunity to dissect the effects attributable to the evolutionary legacy of each genome.

Walunjkar et al. started by producing melting temperature profiles of proteins in both species. As predicted, the large majority of *S. cerevisiae* proteins have higher melting temperatures than orthologs of *S. uvarum*, showing that the *S. cerevisiae* proteome has higher thermal stability of the two species. Then, to isolate the influence of the species’ independent evolutionary history from cellular effects on protein thermal stability, the authors produced first-generation hybrids of the two species. In these hybrids, they performed an ortholog-specific thermal profiling of the proteins to test whether, in a shared cellular environment, *S. cerevisiae* proteins maintained higher thermal stability than their *S. uvarum* orthologs. Their findings indicated that the *S. cerevisiae* proteins were, broadly, still more thermostable; however, the difference was not as elevated as in the parental cells. This reduced difference could be explained by two alternative (and nonmutually exclusive) hypotheses: either the *S. cerevisiae* orthologs had lowered thermal stability in the hybrid cell compared to the parental cell, or the *S. uvarum* orthologs had a relative increase in stability. Their results suggest the latter since, in the hybrid, only *S. uvarum* orthologs had significantly different stability when compared to the parental cells. This process was not associated with any specific group of proteins but reflected instead a general proteome-wide effect.

Proteome analyses thus suggest that both protein structural differences and cellular context contribute to thermal tolerance. The research team then focused on tracing signatures of molecular adaptation at a finer scale; specifically, they tested if *S. cerevisiae* orthologs were also more thermostable when outside the cellular environment. They started by identifying the proteins Guk1 and Aha1 as good candidates for functional testing, since the team had observed consistent melting temperature differences between *S. cerevisiae* and *S. uvarum* alleles, both between parental species and between parental alleles within the hybrid. In vitro assays of these two proteins confirmed that the melting temperature differences between the *S. cerevisiae* and *S. uvarum* orthologs reflected each organism's thermal preferences. However, at the same time, the authors noted that amino acid changes, in isolation, may not necessarily correlate with fitness effects, since *S. cerevisiae* cells expressing a transfected *S. uvarum* Guk1 allele did not show reduced fitness when exposed to different experimental temperatures. Also, although computational predictions of protein stability differences captured the predicted general trend between species, their predicted stability differences were smaller than the differences observed in experimental conditions.

Walunjkar et al. demonstrate how both protein sequence content and cellular environment contribute to the expression of the thermal tolerance phenotype in a complex and interdependent way. These findings have an impact on the general understanding of how organisms adapt to temperature stress. “One broader implication is that protein stability is likely important for even small changes in an organism's thermotolerance,” mentions Fay, who adds that “this may impose a significant constraint on the evolution of thermotolerance, since many proteins would need to be stabilized. However, thermotolerance could also evolve rapidly, if proteins could be stabilized by changes in their cellular environment.” According to the author, future work is needed to distinguish between these distinct evolutionary paths.

**Want to learn more?** Check out these other articles focusing on protein evolution and thermal tolerance recently published in *Molecular Biology and Evolution*:

- “Experimental Evolution in a Warming World: The *Omics* Era” (Santos et al. 2024)
- “Deciphering Structural Traits for Thermal and Kinetic Stability across Protein Family Evolution through Ancestral Sequence Reconstruction” (Cea et al. 2024)
- “The Characterization of Ancient *Methanococcales* Malate Dehydrogenases Reveals That Strong Thermal Stability Prevents Unfolding Under Intense γ-Irradiation” (Madern et al. 2024)

## Data Availability

No data was used in this manuscript.
